# Corticosteroid Biosynthesis Revisited: No Direct Hydroxylation of Pregnenolone by Steroid 21-Hydroxylase

**DOI:** 10.3389/fendo.2021.633785

**Published:** 2021-06-03

**Authors:** Steffen Loke, Anna Stoll, David Machalz, Francesco Botrè, Gerhard Wolber, Matthias Bureik, Maria Kristina Parr

**Affiliations:** ^1^ Pharmaceutical and Medicinal Chemistry, Institute of Pharmacy, Freie Universität Berlin, Berlin, Germany; ^2^ Federazione Medico Sportiva Italiana, Laboratorio Antidoping Federazione Medico Sportiva Italiana (FMSI), Rome, Italy; ^3^ ISSUL—Institute des sciences du sport, Université de Lausanne, Lausanne, Switzerland; ^4^ School of Pharmaceutical Science and Technology, Tianjin University, Tianjin, China

**Keywords:** corticosteroid, cytochrome P450, CYP21A2, GC-MS, fission yeast (*Schizosaccharomyces pombe*), molecular docking, steroid biosynthesis

## Abstract

Cytochrome P450s (CYPs) are an essential family of enzymes in the human body. They play a crucial role in metabolism, especially in human steroid biosynthesis. Reactions catalyzed by these enzymes are highly stereo- and regio-specific. Lack or severe malfunctions of CYPs can cause severe diseases and even shorten life. Hence, investigations on metabolic reactions and structural requirements of substrates are crucial to gain further knowledge on the relevance of different enzymes in the human body functions and the origin of diseases. One key enzyme in the biosynthesis of gluco- and mineralocorticoids is CYP21A2, also known as steroid 21-hydroxylase. To investigate the steric and regional requirements of substrates for this enzyme, we performed whole-cell biotransformation assays using a strain of fission yeast *Schizosaccharomyces pombe* recombinantly expressing CYP21A2. The progestogens progesterone, pregnenolone, and their 17α-hydroxy-derivatives were used as substrates. After incubation, samples were analyzed using gas chromatography coupled to mass spectrometry. For progesterone and 17α-hydroxyprogesterone, their corresponding 21-hydroxylated metabolites 11-deoxycorticosterone and 11-deoxycortisol were detected, while after incubation of pregnenolone and 17α-hydroxypregnenolone, no hydroxylated product was observed. Findings were confirmed with authentic reference material. Molecular docking experiments agree with these results and suggest that interaction between the 3-oxo group and arginine-234 of the enzyme is a strict requirement. The presented results demonstrate once more that the presence of an oxo-group in position 3 of the steroid is indispensable, while a 3-hydroxy group prevents hydroxylation in position C-21 by CYP21A2. This knowledge may be transferred to other CYP21A2 substrates and hence help to gain essential insights into steroid metabolism.

## Introduction

Cytochrome P450 enzymes (CYPs) are an important class of enzymes in the human body. Acting mainly as hydroxylases, they are responsible for a wide variety of metabolic reactions. CYPs form a remarkably versatile enzyme superfamily that has been found in all domains of life and already includes more than 370,000 named members (1). In humans, there are 57 isoforms that can be subdivided by sequence homology into 18 families and 43 subfamilies. However, they may also be categorized according to their subcellular localization, their electron transport chains, or their biological functions (2, 3). On the one hand, substances like xenobiotic drugs can be excreted easier after CYP catalyzed hydroxylation; on the other hand, they also play an essential role in the synthesis of many endogenous compounds (4). Six of these enzymes are reported to fulfill critical physiological functions in steroid hormone biosynthesis, namely CYP11A1 (cytochrome P450 scc, side-chain cleavage enzyme), CYP17A1 (cytochrome P450 17α, steroid 17α-hydroxylase and 17, 20-lyase), CYP21A2 (cytochrome P450 c21, steroid 21-hydroxylase), CYP11B1 (cytochrome P450 11β, steroid 11β-hydroxylase), CYP11B2 (cytochrome P450 aldo, aldosterone synthase) and CYP19A1 (cytochrome P450 aro, aromatase) (5).

Steroidogenic CYPs display high substrate specificity, which only allows a small group of substances with a defined structure to be transformed. This regio- and stereospecific oxy functionalization is of high biological relevance, and especially CYPs involved in steroid biosynthesis have been considered to be unable to metabolize xenobiotics. In the last decade, this hypothesis, however, was demonstrated to be not correct (6–10). Still, strict structural requirements for substrates have been demonstrated. As recently reported, substrates of CYP21A2 require a 3-oxo functionality at the steroid A-ring to be successfully hydroxylated at the side chain attached to the D-ring (7).

CYP21A2 is an important enzyme in the biotransformation of progestogens into corticosteroids. The enzyme converts the endogenous progestogens progesterone (pregn-4-ene-3,20-dione, PRO) and 17α-hydroxyprogesterone (17α-hydroxypregn-4-ene-3,20-dione, 17αOH-progesterone, 17PRO) to 11-deoxycorticosterone (21-hydroxypregn-4-ene-3,20-dione, DOC) and 11-deoxycortisol (17α,21-dihydroxypregn-4-ene-3,20-dione, RSS), respectively, by hydroxylation at the C-21 position (11). Therefore, it plays an essential role in the biosynthesis of both gluco- and mineralocorticoids. Hence, humans with reduced activity of CYP21A2 may suffer from congenital adrenal hyperplasia (CAH), which is characterized by decreased production of cortisol (12). Due to feedback mechanisms of the hypothalamic-pituitary-adrenal axis, increased concentrations of progestogens, but also of androgens resulting from alternative metabolic pathways, are observed in these patients (13).

Based on recent findings on substrate selectivity of human CYP21A2, this work gives a deeper insight into the endogenous compounds PRO, 17PRO, pregnenolone (PRE), and 17α-hydroxypregnenolone (17PRE). These substances have been reported as substrates of the enzyme in the KEGG database and by Cathro et al. (14). The data presented in this work help to extrapolate findings to other potential substrates of CYP21A2 and may thus help to identify new metabolites or inhibitors of the enzyme.

## Materials and Methods

### Chemicals and Reagents

The substrates PRO, PRE, 17PRO, and 17PRE as well as the analytical references DOC and RSS, were obtained from Steraloids Inc. (Newport, Rhode Island, USA). *N*-Methyl-*N*-(trimethylsilyl)trifluoro acetamide (MSTFA) was purchased from Chemische Fabrik Karl Bucher GmbH (Waldstetten, Germany). All other chemicals and solvents were purchased from AppliChem GmbH (Darmstadt, Germany), Merck (Darmstadt, Germany), Karl Roth (Karlsruhe, Germany), Sigma Aldrich (Steinheim, Germany), Thermo Fischer (Karlsruhe, Germany), or VWR International (Darmstadt, Germany).

### GC-MS Analyses

Gas chromatographic-mass spectrometric (GC–MS) analysis of the samples was performed on an Agilent 7890A gas chromatographic system coupled to an Agilent 5975 C inert mass selective detector equipped with an Agilent HP1 column (17 m, 0.2 mm id, 0.11 μm film thickness). The following parameters were used for the analysis of products: carrier gas: helium, oven program: 183 °C, +3 °C/min to 232 °C (rate 1), +40 °C/min to 310 °C (rate 2), hold for 2 min, injection volume: 2 μL, split 16:1, injection temperature: 300 °C, electron ionization (EI): 70 eV, full scan mode from m/z 40 to m/z 1000. Prior to GC–MS analysis the dried residues were pertrimethylsilylated (TMS derivatives) by reaction with 100 μL TMIS reagent (MSTFA/ammonium iodide/ethanethiol, 1000:2:3, v:w:v) at 75 °C for 20 min.

### Steroid 21-Hydroxylation Assay

All experiments were conducted using the recombinant fission yeast strain CAD75. As reported earlier, it co-expresses the recombinant human steroid 21-hydroxylase together with human cytochrome P450 reductase (CPR) (genotype: *h- ura4-D.18 leu1:: pCAD1-hCPR/pNMT1-CYP21A2*) (15). Whole-cell biotransformation assays were performed as described before (7). In brief, cells were cultured for three days at 30 °C on plates containing 5 µM thiamin. Pre- and main cultures were prepared in Edinburgh Minimal Medium (EMM) (8). Substrates, dissolved in ethanol (PRO, PRE, 17PRO) or methanol/dimethyl sulfoxide (1 + 1 v/v) (17PRE), were added in a concentration of 500 µM to cell suspension of CAD75 fission yeast in EMM and incubated for 72 h at 30 °C under mild agitation. The cofactor NADPH (needed for the P450-dependent bioconversion) is provided by the fission yeast cells itself in sufficient quantity (16). After centrifugation and separation from the cell pellet, the supernatant was extracted with ethyl acetate. The organic phase was dried, and product analysis was performed by GC-MS after trimethylsilyl-derivatization, as described in the section *GC-MS Analyses*.

### Molecular Modeling

Molecular docking experiments were performed using GOLD software [Genetic Optimisation for Ligand Docking, The Cambridge Crystallographic Data Centre, UK (17, 18) v.5.2] with 10 genetic algorithm (GA) runs. 3D starting geometries for substrates PRE, 17PRO, and 17PRE were obtained with CORINA [3D Structure Generator CORINA Classic, Molecular Networks GmbH, Nuremberg, Germany (19)]. The X-ray structure of CYP21A2 co-crystallized with PRO [PDB: 4Y8W (20)] served as protein conformation. The binding pocket was defined by a sphere of 12 Å radius with the C-8 atom of PRO as its center. GOLD was executed with standard settings, besides setting search efficiency to 200% and enabling the ‘Generate diverse solutions’ option. Key binding site residue Arg234 was marginally rotated during its energy minimization conducted in the MOE software package (Molecular Operating Environment 2019.01; Chemical Computing Group ULC, Montreal, Canada) to allow for optimal hydrogen bonding to 17PRE. This Arg234 orientation was kept for all other compounds. Subsequently, energy minimization of and 3D pharmacophore modeling for the obtained poses was conducted in LigandScout (21, 22) v.4.2. using the MMFF94 force field (23).

## Results

### Steroid 21-Hydroxylation Assay

**Figure 1:**
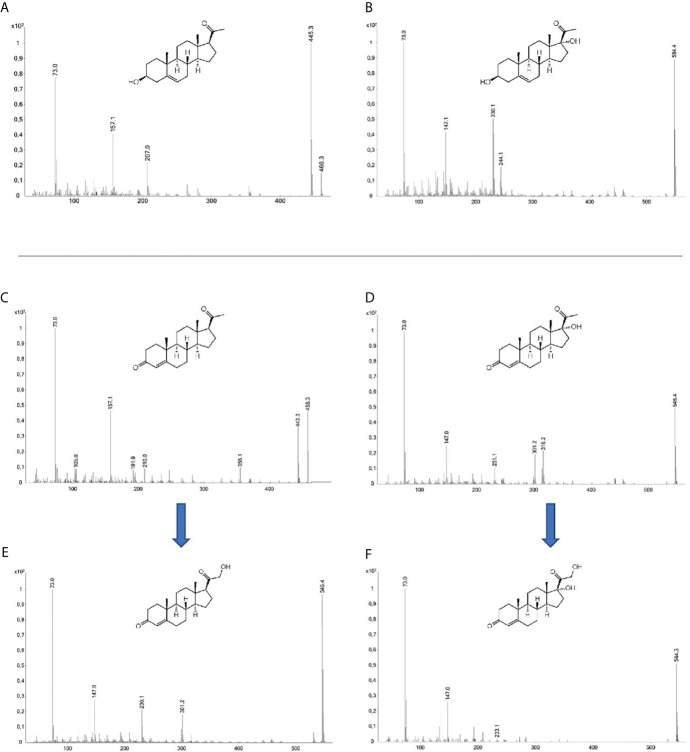
Mass spectra (GC-EI-MS) of the TMS derivatives of pregnenolone **(A)**, 17α-hydroxypregnenolone **(B)**, progesterone **(C)**, 17α-hydroxyprogesterone **(D)**, 11-deoxycorticosterone **(E)**, 11-deoxycortisol **(F)**; abscissa: m/z, ordinate. intensity normalized to the highest peak.

The substrates PRO, PRE, 17PRO, and 17PRE, were incubated with steroid 21-hydroxylase in a whole-cell biotransformation assay. After work-up, the GC-MS analysis of the incubation broth showed characteristic peaks for the 21-hydroxylated products of PRO and 17PRO. The product of PRO showed peaks at m/z 546 and a loss of 245, the product of 17PRO showed an abundant peak at m/z 544 and a subsequent loss of 311. The corresponding mass spectra of the TMS derivatives are presented in **Figures 1E, F**. Additionally, peaks of the remaining substrate were present in the chromatograms as well (mass spectra in **Figures 1C, D**). Product structures were deduced from the mass spectra from fragmentation analysis and later confirmed by comparison with an authentic reference material of DOC and RSS analyzed by GC-MS in parallel. Similar retention times and characteristic fragment ions were obtained for both, reference and products of hydroxylation.

**Figure 2:**
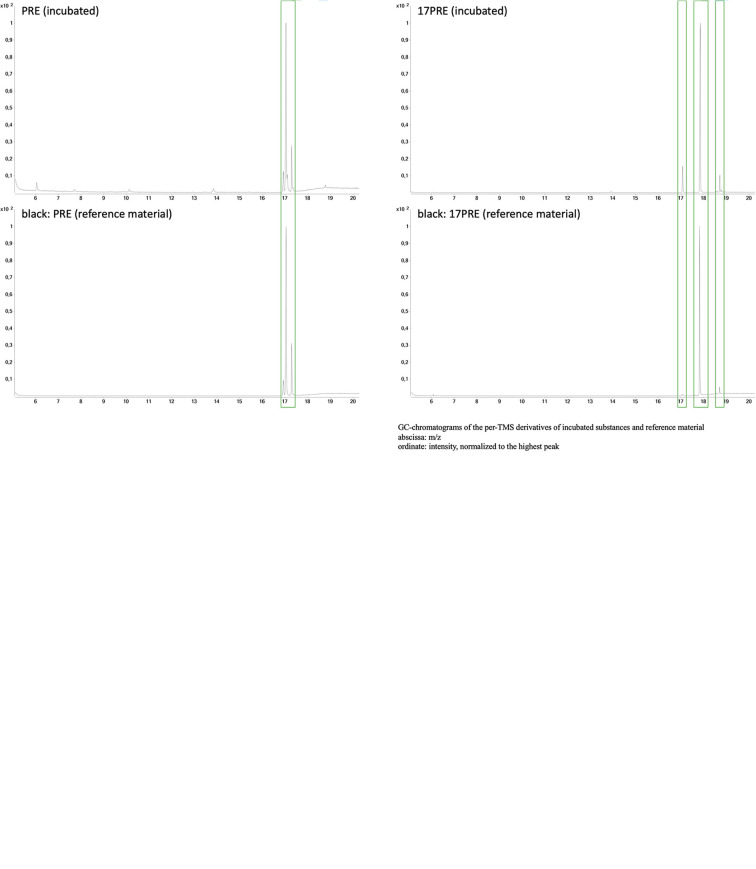
Upper two rows: Chromatograms (GC-EI-MS) of the TMS derivatives of progesterone (PRO), 17-hydroxyprogesterone (17PRO), and their hydroxylated products after incubation in comparison to the authentic reference material (due to the formation of derivatization isomers, multiple peaks are obtained for one analyte). Lower two rows: Chromatograms (GC-EI-MS) of the TMS derivatives of pregnenolone (PRE) and 17-hydroxypregnenolone (17PRE) after incubation in comparison to the authentic reference material (due to the formation of derivatization isomers, multiple peaks are obtained for one analyte).

In contrast, no hydroxylated substrate was detectable after incubation of PRE or 17PRE. Mass spectra of substrates are displayed in **Figures 1A, B**. Comparison of the chromatograms revealed no product formation for these two substrates. The chromatograms of the incubated substances in comparison with the authentic reference material are displayed in **Figure 2**. As reported earlier, multiple peaks may be detected for one analyte due to the formation of derivatization isomers (24).

### Modeling Binding of 17PRO, PRE and 17PRE to Steroid 21-Hydroxylase

**Figure 3:**
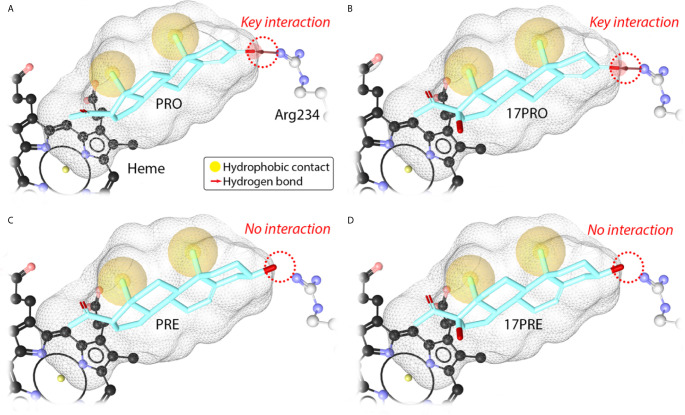
Substrate binding to the active site of CYP21A2. Progesterone binding mode from X-ray structure (PDB: 4Y8W) (**A**, PRO). Suggested binding modes of 17α-hydroxyprogesterone (**B**, 17PRO), pregnenolone (**C**, PRE) and 17α-hydroxypregnenolone (**D**, 17PRE) obtained from docking. Active substrates **(A, B)** form the key interaction to Arg234. Inactive substrates **(C, D)** do not show this interaction, which makes productive binding highly unlikely.

In order to rationalize the observed differences in the 21-hydroxylation activity of CYP21A2, docking experiments for PRE, 17PRO and 17PRE were performed based on the X-ray structure of CYP21A2 co-crystallized with PRO [PDB: 4Y8W (20), **Figure 3**]. The catalytically competent binding mode of PRO present in the X-ray structure places the C-21 atom 4 Å away from the heme iron. The hydrogen bond of Arg234 and the oxo group at C-3 of the substrate is critical to catalytically competent binding (7) (**Figure 3A**). According to the pharmacophore model the substrate methyl groups of PRO and 17PRO form hydrophobic contacts to the active site of CYP21A2. The key hydrogen bond to Arg234 is also present in the pharmacophore model of the most plausible docking pose of 17PRO, which is highly similar to the orientation of PRO (**Figure 3B**). The C-21 atoms of PRO and 17PRO are in a highly similar position (4 Å away from the heme) and hence the 17PRO pose is deemed catalytically competent as well.

In contrast, the most plausible docking poses of PRE and 17PRE corresponding to catalytically competent orientation do not involve hydrogen bonding to key residue Arg234 (**Figures 3C, D**), because the hydroxy groups at C3 represent only very weak hydrogen bond acceptor properties. We suggest that these orientations and productive binding of PRE or 17PRE to steroid 21-hydroxylase (CYP21A2) does not occur *in vivo*. Hence, we further suggest that conversion of PRE and 17PRE to 21-hydroxypregnenolone (T) and 17, 21-dihydroxypregnenolone (R), respectively, is highly unlikely.

## Discussion

**Figure 4:**
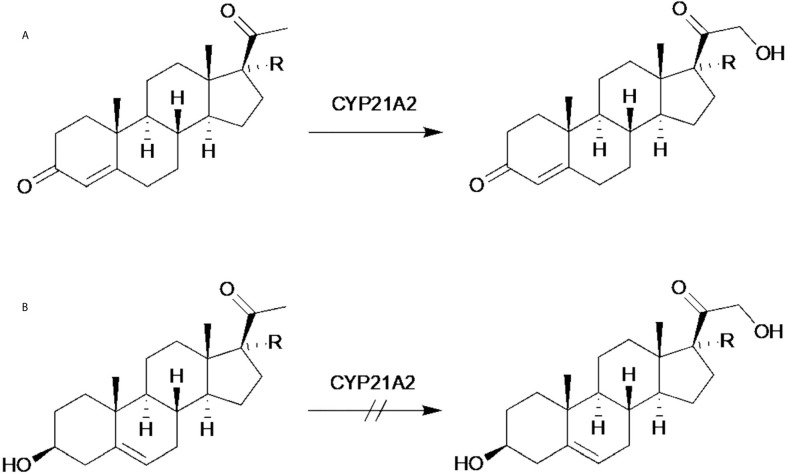
A-ring structure dependent hydroxylation of progestogens **(A)** successful hydroxylation of 3-keto steroids (R = H = progesterone; R = OH = 17α-hydroxyprogesterone), **(B)** no hydroxylation of 3-hydroxy steroids (R = H = pregnenolone; R = OH = 17α-hydroxypregnenolone).

As expected, PRO and 17PRO are substrates for 21-hydroxylation catalyzed by human CYP21A2 (**Figure 4A**). The resulting products were confirmed as DOC and RSS by GC-MS comparison with authentic reference material [confidence level 1, according to Schymanski et al. (25)]. GC-MS analysis showed a characteristic loss of 245 for DOC corresponding to a fragment of the hydroxylated D-ring (**Figure 1E**). For RSS, no peak for the molecular ion (m/z 634) could be detected (**Figure 1F**). However, a signal with m/z 544 was observed, which is identified as [M-90]^+^ (a loss of TMSOH), and a subsequent loss of 311, resulting in a fragment with m/z 233, which is caused by B/C-ring fragmentation.

The rates of successful hydroxylation in the assays were calculated by comparison of the areas in the GC-chromatograms. After 72 h PRO was hydroxylated to 92%, 17PRO was hydroxylated to over 99%. In a previous publication the product space-time yields for the conversion of PRO (1.6 ± 0.5 μmol/g/day) and 17PRO (2.1 ± 0.7 μmol/g/day) by whole-cell biotransformation with strain CAD75 were reported (15). The absence of any signal for potential products of hydroxylation in case of PRE and 17PRE substantiates the hypothesis of no hydroxylation in the latter cases.

These results are in line with common knowledge of steroid biosynthesis, where the progestogens PRO and 17PRO are converted to corticoids (5) by 21-hydroxylation. Similarly, Lattemann et al. (26) reported the successful 21-hydroxylation of 3-keto steroids in whole-cell biotransformation utilizing human CYP21A2 recombinantly expressed in *E. coli*. They disclosed the successful conversion of natural and non-natural steroid substrates, all showing a 3-oxo group. As demonstrated in earlier experiments, the A-ring composition of CYP21A2-substrates plays a crucial role in hydroxylation. It was demonstrated that the 3-oxo group is a strict prerequisite for CYP21A2 substrates (7). In line with these findings, the incubation of endogenous 3-hydroxy steroids PRE and 17PRE did not lead to their 21-hydroxylated analogs (**Figure 4B**). No signals corresponding to hydroxylated metabolites of PRE or 17PRE were detected following their incubation with CYP21A2. Molecular docking experiments supported the structure requirements. Interaction of the carbonyl group at C-3 of the substrate to the side-chain Arg234 of the enzyme is indispensable.

**Figure 5:**
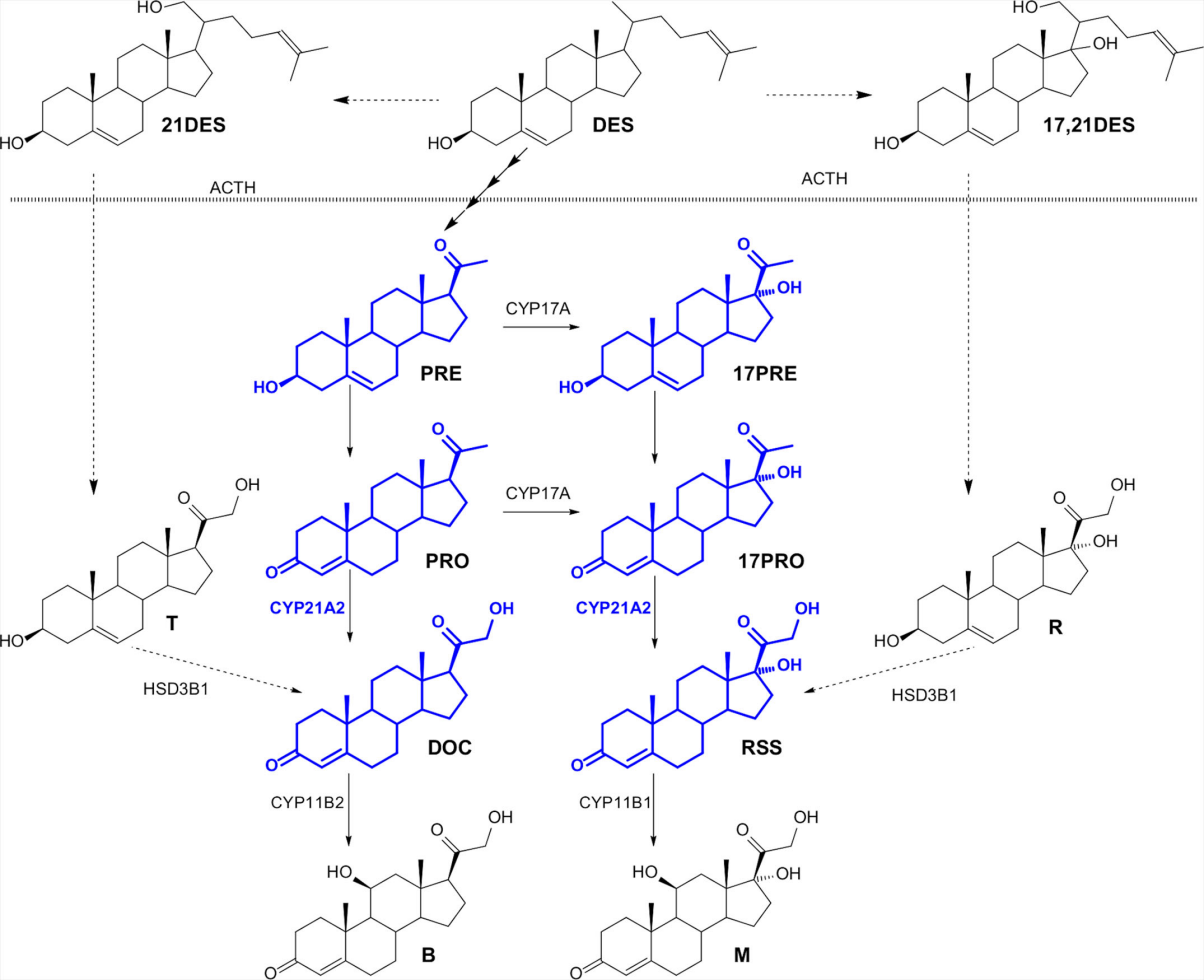
Hypothesized corticosteroid biosynthesis, adapted from Kaufmann et al. (31) steroid precursor desmosterol (DES) is converted *via* different pathways to corticosterone (B) and cortisol (M) involving 21-hydroxydesmosterol (21DES), 17,21-dihydroxydesmosterol (17,21DES), pregnenolone (PRE), 17-hydroxypregnenolone (17PRE), progesterone (PRO), 17-hydroxyprogesterone (17PRO), 11-deoxycorticosterone (DOC), 21-hydroxypregnenolone (T), 11-deoxycortisol (RSS) and 17,21-dihydroxypregnenolone (R).

In contrast, earlier reports from literature postulate the 21-hydroxylation of PRE based on *in vitro* experiments utilizing adrenal microsomes (27). Human adrenal microsomes yielded R after incubation of PRE in their assay. Already reported in the 1960s, PRE is transformed into PRO prior to 21-hydroxylation (28). Significant amounts of T *in vivo* are reported in newborn infants (day 0-3 after delivery) or adult males after corticotrophin stimulation, while generally only small amounts of T were detected in adult humans (29, 30). Cathro et al. thus postulated the direct 21-hydroxylation as a subsidiary pathway in humans (30). Furthermore, Cathro et al. (14) postulated the direct 21-hydroxylation of PRE, while 17PRE is reported to undergo side-chain cleavage to yield dehydroepiandrosterone (DHEA). However, in 1980 Kaufmann et al. hypothesized that alternative pathways of corticosteroid biosynthesis exist. As postulated, T, and R are most likely generated *via* 21-hydroxy- or 17,21-dihydroxydesmosterol involving adrenocorticotropin (ACTH) rather than direct hydroxylation of PRE or 17PRE (31). According to our results we consider it likely that also the 21-hydroxylation of desmosterol is not catalyzed by steroid 21-hydroxylase in humans (**Figure 5**). The presented results hence help to better understand corticoid formation and may thereby provide important basic knowledge for the treatment of diseases that are linked to enzyme deficiencies involved in corticoid biosynthesis.

## Data Availability Statement

The raw data supporting the conclusions of this article will be made available by the authors, without undue reservation.

## Author Contributions

Conceptualization, MB and MP. *In vitro* methodology and data analysis, SL, AS, MB, and MP. *In silico* methodology and analysis, DM and GW. Resources, GW, MB, and MP. Data curation, SL, AS, and DM. Writing—original draft preparation, SL and MP. Writing—review and editing, AS, DM, GW, FB, and MB. Visualization, SL, AS, and DM. Supervision, GW and MP. Project administration, MP. Funding acquisition, FB, MB, and MP. All authors have read and agreed to the published version of the manuscript. All authors contributed to the article and approved the submitted version.

## Funding

This study was partially funded by the World Anti-Doping Agency (WADA) within WADA 15A21MP.

## Conflict of Interest

The authors declare that the research was conducted in the absence of any commercial or financial relationships that could be construed as a potential conflict of interest.
